# Metformin Restores the Drug Sensitivity of MCF-7 Cells Resistant Derivates via the Cooperative Modulation of Growth and Apoptotic-Related Pathways

**DOI:** 10.3390/ph13090206

**Published:** 2020-08-21

**Authors:** Danila Sorokin, Yuri Shchegolev, Alexander Scherbakov, Oxana Ryabaya, Margarita Gudkova, Lev Berstein, Mikhail Krasil’nikov

**Affiliations:** 1 Department of Experimental Tumor Biology, Institute of Carcinogenesis, N.N. Blokhin National Medical Research Center of Oncology of the Ministry of Health of Russia, Moscow 115522, Russia; dsorokin2018@gmail.com (D.S.); yurashhegolev@gmail.com (Y.S.); gudkova@ronc.ru (M.G.); krasilnikovm1@yandex.ru (M.K.); 2 Department of Experimental Diagnostic and Tumor Therapy, N.N. Blokhin National Medical Research Center of Oncology of the Ministry of Health of Russia, Moscow 115522, Russia; oxa2601@yandex.ru; 3 Scientific Lab of Subcellular Technologies with the Group of Oncoendocrinilogy, N.N. Petrov National Medical Research Center of Oncology, Saint Petersburg 197758, Russia; levmb@endocrin.spb.ru

**Keywords:** metformin, resistance, cancer, AP-1—the transcription factor activator protein 1 (IPR000837), NF-κB—the nuclear factor kappa-light-chain-enhancer of activated B cells (IPR030495), breast cancer, signaling pathways

## Abstract

The phenomenon of the primary or acquired resistance of cancer cells to antitumor drugs is among the key problems of oncology. For breast cancer, the phenomenon of the resistance to hormonal or target therapy may be based on the numerous mechanisms including the loss or mutation of estrogen receptor, alterations of antiapoptotic pathways, overexpression of growth-related signaling proteins, etc. The perspective approaches for overcoming the resistance may be based on the usage of compounds such as inhibitors of the cell energetic metabolism. Among the latter, the antidiabetic drug metformin exerts antitumor activity via the activation of AMPK and the subsequent inhibition of mTOR signaling. The experiments were performed on the ERα-positive MCF-7 breast cancer cells, the MCF-7 sublines resistant to tamoxifen (MCF-7/T) and rapamycin (MCF-7/Rap), and on triple-negative MDA-MB-231 breast cancer cells. We have demonstrated metformin’s ability to enhance the cytostatic activity of the tamoxifen and rapamycin on both parent MCF-7 cells and MCF-7-resistant derivates mediated via the suppression of mTOR signaling and growth-related transcriptional factors. The cooperative effect of metformin and tested drugs was realized in an estrogen-independent manner, and, in the case of tamoxifen, was associated with the activation of apoptotic cell death. Similarly, the stimulation of apoptosis under metformin/tamoxifen co-treatment was shown to occur in the MCF-7 cells after steroid depletion as well as in the ERα-negative MDA-MB-231 cells. We conclude that metformin co-treatment may be used for the increase and partial restoration of the cancer cell sensitivity to hormonal and target drugs. Moreover, the combination of metformin with tamoxifen induces the apoptotic death in the ERα-negative breast cancer cells opening the additional perspectives in the treatment of estrogen-independent breast tumors.

## 1. Introduction

Breast cancer is one of the main causes of mortality from cancers. Considering the high social significance of this disease in the majority of scientific centers, development is being made to improve early diagnostics and search for new methods of breast cancer treatment. Estrogens are the key regulators for breast cell metabolism in health and disease. According to the results of different epidemiologic investigations, approximately 70% of breast tumors in postmenopausal females have steroid hormone receptors, which makes the use of hormonal and antihormonal drugs possible [1,2]. Antiestrogen tamoxifen has been used in clinical practice since the 1970s and remains the “gold standard” of hormone therapy [3].

Estrogen receptors (ERα) and progesterone receptors (PR) are validated markers of tumor cells’ hormone sensibility in clinical practice. It is critically important that more than 70% of patients with positive receptor status (ERα+, PR+) are initially susceptible to tamoxifen therapy; generally, in this group, there is a high percentage of patients demonstrating objective response to the drug during adjuvant and neoadjuvant treatment of breast cancer. Hormonal therapy may represent a challenge due to different types of resistance that develop de novo or during the use of hormonal drugs. In the past 20 years, intense investigations have been carried out to elucidate signal pathways in tumor cells responsible for hormone resistance development. It has been stated that long-term exposure to hormone drugs causes the rearrangement of intracellular pathways and excessive activation of growth factor-dependent signaling, among the latter: EGFR/HER2, IGFR, PI3K/Akt/mTOR, etc. In the setting of high activity of growth factors, signaling tamoxifen loses its efficacy which causes the disease relapse [4,5,6].

Target therapy belongs to the most effective approaches to treat the resistant breast tumors directly blocking the hyper-expressed signaling proteins, e.g., tyrosine kinases (HER2/Neo, EGFR), Akt, mTOR, and some others [7]. However, similar to endocrine therapy, acquired resistance to target drugs remains among the main factor limiting the effectiveness of targeted anticancer therapy [8,9,10]. mTOR inhibitors (rapamycin and its analogs also known as rapalogs) belong to the target drugs that are widely used in the treatment of the different types of cancer including breast cancer. The resistance to mTOR inhibitors is accompanied by the hyperexpression of various cancer-associated signaling proteins, among them PI3K, Akt. Ras, etc., preventing and/or attenuate the cytotoxic drug action [11,12].

Numerous studies have shown that metformin, a well-known antidiabetic drug, exerts the antitumor activity on the different types of cancer, both in vivo and in vitro [13,14]. The diversified effects of metformin affect various cell functions, including inhibition of gluconeogenesis and oxidative phosphorylation, affecting cell mobility, cell growth, autophagy, etc. [15,16]. The mechanism of the antitumor metformin activity was found to be mediated, at least in a part, via the activation of AMPK with following suppression of mTOR signaling [17,18,19]. Single observations demonstrate the metformin ability to enhance the cytotoxic action of antitumor drugs [20,21,22,23]; however, the mechanism of these effects, as well as the dependence on drug combination, are still unclear.

Here, we found that a combination of metformin with tamoxifen or rapamycin increases the sensitivity of breast cancer cells to the respective drugs. The potentiating metformin action was shown to occur via the additional inhibition of mTOR signaling as well as the suppression of the growth-related transcriptional factors. The anti-growth effect of co-treatment of metformin with tamoxifen or rapamycin was realized in an estrogen-independent manner, and, in the case of tamoxifen, was associated with the activation of apoptotic cell death.

## 2. Results

### 2.1. Metformin and MCF-7 Cells Response to Cytostatic Drugs

**Figure 1:**
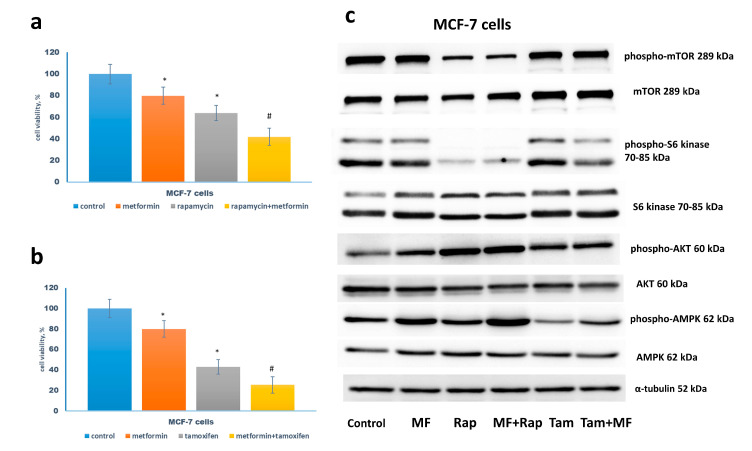
Drug sensitivity of MCF-7 cells. (**a**,**b**) Cell growth. The cells were treated without or with 1 µM rapamycin and/or 2 mM metformin (**a**), 5 µM tamoxifen and/or 2 mM metformin (**b**) for 3 days in standard DMEM medium with 10% FBS, and the amount of the viable cells was counted by the MTT-test; Data represent mean value ± SD of three independent experiments. One-hundred percent was set as the viability of cells treated with vehicle control. *p* < 0.05: * versus control, # versus either drug alone and control. (**c**) Western blot analysis. The MCF-7 cells were treated as indicated above. Western blot analysis of AMPK, phospho-AMPK, mTOR, phospho-mTOR, S6 kinase, phospho-S6 kinase, Akt, phospho-Akt was performed in the MCF-7 cell extracts. Protein loading was controlled by membrane hybridization with α-tubulin antibodies.

**Figure A1:**
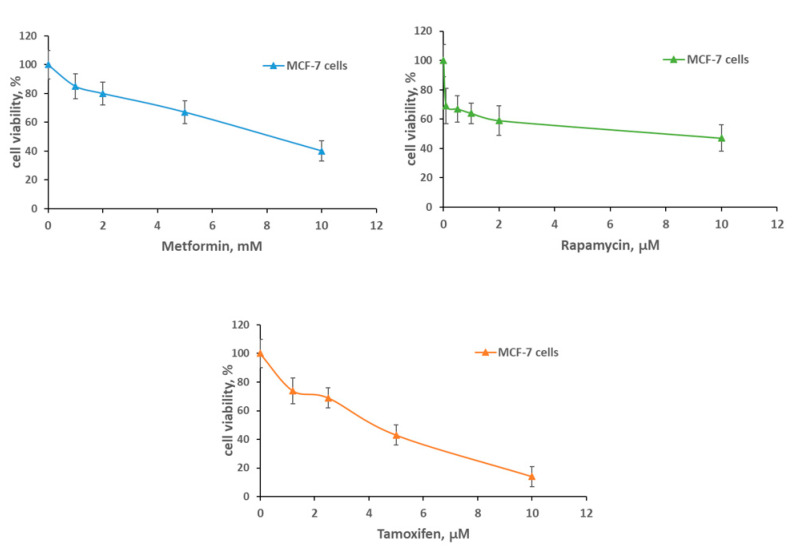
Dose-response curves for metformin, rapamycin, and tamoxifen. MCF-7 cell viability was assessed by the MTT test after 72h growth with compounds. The IC_50_ values were 5.8 mM for metformin and 4.1 µM for tamoxifen. The IC_50_ calculation for rapamycin was not validated due to the plateau curve.

At the first stage of the experiments, the efficiency of the growth inhibitory effect of metformin in the combination with various cytostatic drugs was compared. Among the latter substances were rapamycin, direct mTOR inhibitor, and tamoxifen, antiestrogen drug affecting estrogen-dependent growth signaling. The dose-response curves for tamoxifen and metformin had standard shapes, whereas, for rapamycin, the curve had a long plateau (Figure A1). This characteristic of rapamycin was discussed earlier in the works [24,25]. The analysis of the MCF-7 cell response to drug combination revealed additive effects for tamoxifen and metformin treatments. Similarly, metformin increased the efficiency of rapamycin action, but with a lesser extent (Figure 1a,b).

The study of the mTOR signaling revealed the marked suppression of the phosphorylation of S6 kinase by rapamycin or tamoxifen in the combination with metformin that correlated with metformin-induced AMPK phosphorylation (Figure 1c). Importantly, S6 kinase suppression was accompanied by Akt activation supporting the existence of the well-described negative feedback between Akt and mTOR signaling [26].

**Figure 2:**
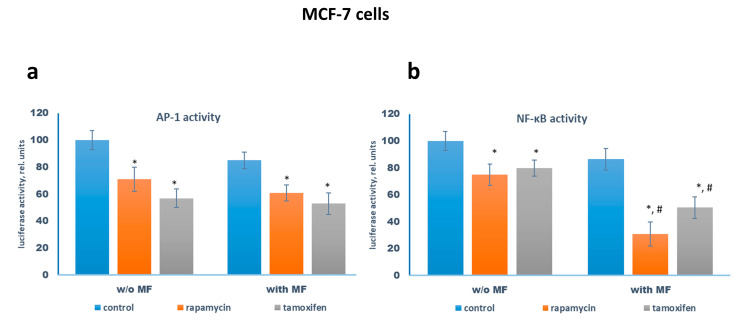
Reporter analysis of the transcriptional activity of AP-1 and NF-κB in the MCF-7 cells. The MCF-7 cells were pretreated with or without 2 mM metformin for 2 days, then the cells were transfected with the AP-1 (**a**) or NF-κB (**b**) plasmid containing the luciferase reporter gene under the AP-1 or NF-κB-responsive elements, respectively, and β-galactosidase plasmid. Three hours after transfection the cells were treated with or without 1 µM rapamycin, 2 mM metformin (MF), and 5 µM tamoxifen for 24 h. The luciferase and β-galactosidase activities were determined as described in Materials and Methods. The relative luciferase activity was calculated in arbitrary units as the ratio of the luciferase to the galactosidase activity. Data represent the mean value ± SD of three independent experiments. *p* < 0.05: * versus respective control, # versus respective probes w/o metformin.

Reporter analysis of the transcriptional activity of AP-1 and NF-κB showed the suppression of it by rapamycin or tamoxifen. Metformin alone exhibited a slight inhibitory effect, whereas the combination of metformin with rapamycin or tamoxifen resulted in the additional suppression of NF-κB demonstrating metformin ability to potentiate the anti-growth activity of both drugs (Figure 2).

### 2.2. Metformin Increases the Sensitivity to Treatment of MCF-7 Cells Resistant Derivates

**Figure 3:**
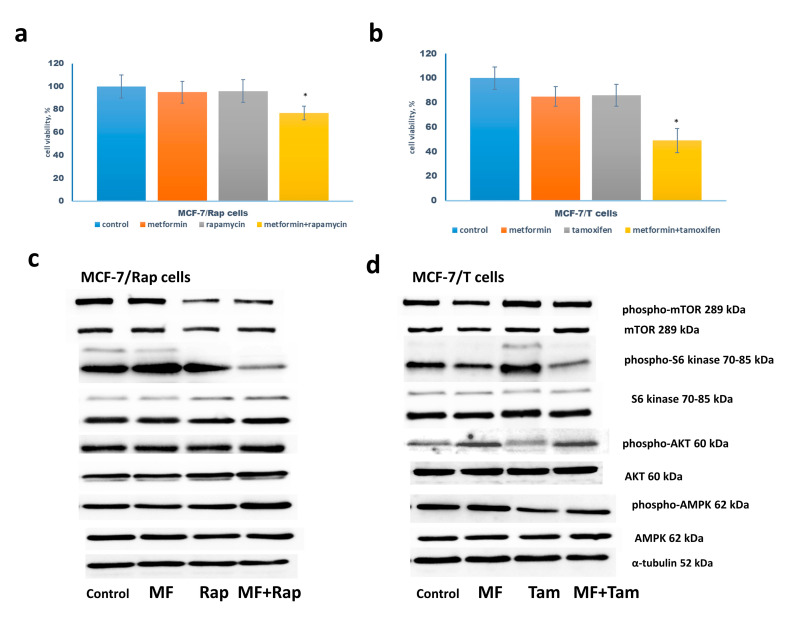
Drug sensitivity of MCF-7/Rap and MCF-7/T cells. (**a**,**b**) Cell growth. The MCF-7/Rap cells were treated without or with 1 µM rapamycin in the presence or absence 2 mM metformin (MF) (a), the MCF-7/T cells—with 5 µM tamoxifen (Tam) and/or 2 mM metformin (b) for 3 days in standard DMEM medium with 10% FBS, and the amount of the viable cells was counted by the MTT test; Data represent mean value ± SD of three independent experiments. One-hundred percent was set as the viability of cells treated with vehicle control. *p* < 0.05: * versus control and either drug alone. (**c**,**d**) Western blot analysis. The MCF-7/Rap and MCF-7/T cells were treated as indicated above. Western blot analysis of AMPK, phospho-AMPK, mTOR, phospho-mTOR, S6 kinase, phospho-S6 kinase, Akt, phospho-Akt was performed in the cell extracts. Protein loading was controlled by membrane hybridization with α-tubulin antibodies.

**Figure 4:**
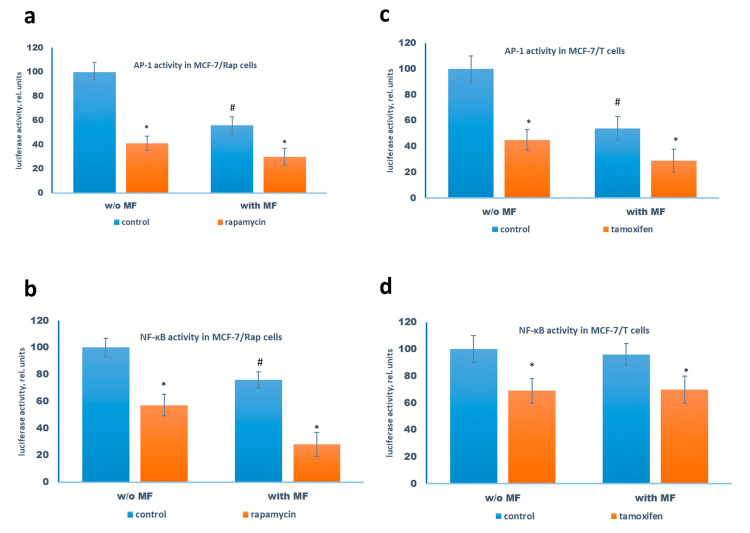
Reporter analysis of the transcriptional activity of AP-1 and NF-κB in the MCF-7/Rap and MCF-7/T cells. The MCF-7/Rap (**a**,**b**) and MCF-7/T (**c**,**d**) cells were pretreated with or without 2 mM metformin for 2 days, then the cells were transfected with the AP-1 (**a,c**) or NF-κB (**b,d**) plasmid containing the luciferase reporter gene under the AP-1 or NF-κB-responsive elements, respectively, and β-galactosidase plasmid. Three hours after transfection the cells were treated with 2 mM metformin (MF) and 1 µM rapamycin (**a,b**) or 2 mM metformin and 5 µM tamoxifen (**c,d**) for 24 h. The luciferase and β-galactosidase activities were determined as described in Materials and Methods. *p* < 0.05: * versus respective control, # versus control cells w/o metformin. Data represent the mean value ± SD of three independent experiments.

The following experiments were performed on the rapamycin-resistant MCF-7/Rap cells developed by the long-term treatment of the parent cells with increased doses of rapamycin, and tamoxifen-resistant MCF-7/T cells obtained by continuous tamoxifen treatment. The combination of metformin with rapamycin or tamoxifen was found to increase the sensitivity of the resistant cells to respective drugs (Figure 3a,b). The study of the mTOR signaling pathway revealed the metformin-induced changes in the signaling proteins similar to that in the parent MCF-7 cells: additional suppression of the S6 kinase phosphorylation by rapamycin or tamoxifen that correlated with metformin-induced AMPK phosphorylation (Figure 3c,d). The reporter analysis of AP-1 and NF-κB transcriptional activity showed marked AP-1 inhibition by the combination of metformin with rapamycin or tamoxifen in both resistant cells, and NF-κB suppression in the MCF-7/Rap cells (Figure 4).

The data presented demonstrate that metformin-induced additional suppression of mTOR signaling may be considered as one of the factors responsible for the metformin ability to potentiate the cytotoxic action of the target drugs.

### 2.3. Metformin and Estrogen Signaling

**Figure 5:**
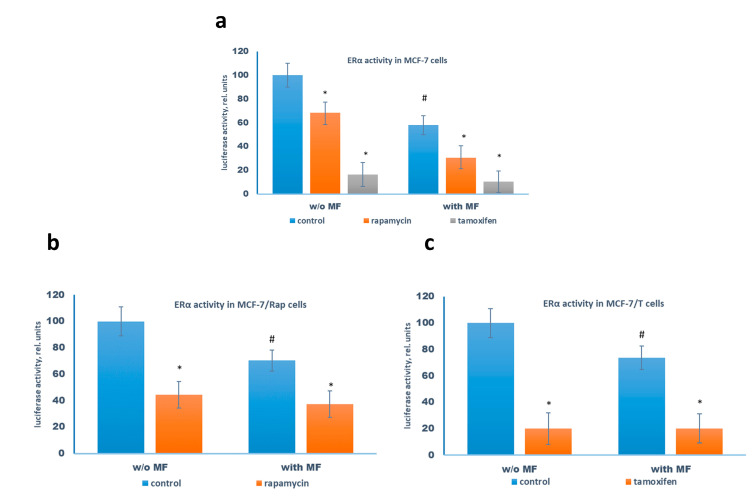
Reporter analysis of the transcriptional activity of the estrogen receptor. The MCF-7 (**a**), MCF-7/Rap (**b**), and MCF-7/T (**c**) cells were pretreated with or without 2 mM metformin (MF) for 2 days, then the cells were transfected with the ERE plasmid containing the luciferase reporter gene under the estrogen-responsive element (ERE) and β-galactosidase plasmid. After 3 h the cells were treated for 24 h with the combination of tested drugs as indicated at the diagram. The luciferase and β-galactosidase activities were determined as described in Materials and Methods. Data represent the mean value ± SD of three independent experiments. *p* < 0.05: * versus respective control, # versus control cells w/o metformin.

Earlier we and others demonstrated the metformin ability to induce the partial inhibition of estrogen receptor (ERα) activity in the MCF-7 cells [18,27,28]. To elucidate the role of ERα signaling in the transferring of metformin effects, the influence of the cytostatic drugs and metformin on the ERα activity was studied. We found that metformin treatment inhibited ERα transcriptional activity both in the parent MCF-7 cells and in the resistant sublines. As expected, the cell treatment with tamoxifen, alone or in the combination with metformin, resulted in the significant suppression of the ERα transcriptional activity in the treated cells; furthermore, the rapamycin treatment caused the remarkable suppression of ERα activity, probably as the result of the rapamycin-induced suppression of the protein translation machinery (Figure 5).

**Figure 6:**
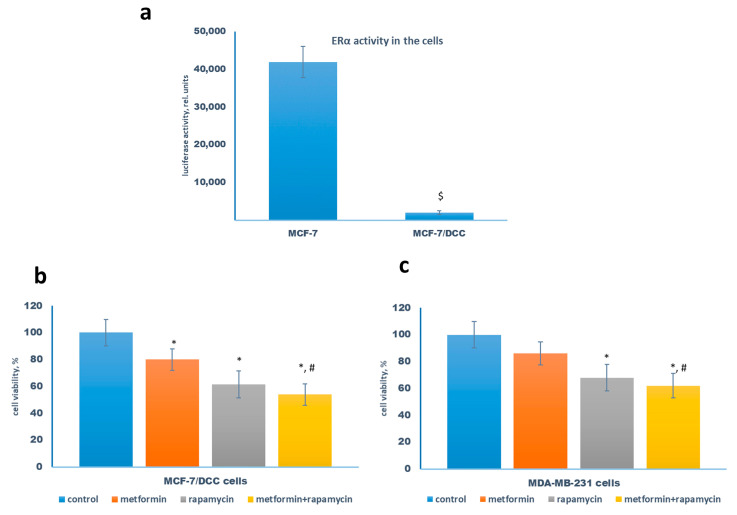
ERα inactivation and cell sensitivity to rapamycin. (**a**) The comparative analysis of the transcriptional activity of the estrogen receptor in MCF-7 cells and MCF-7/DCC cells, pretreated in the steroid-depleted medium for 3 days. The ERE luciferase and β-galactosidase activities were determined as described in Materials and Methods. Data represent the mean value ± SD of three independent experiments. (**b**,**c**) The sensitivity of MCF-7/DCC cells and ERα-negative MDA-MB-231 cells to metformin and rapamycin. The cells were treated without or with 1 µM rapamycin in the presence or absence of 2 mM metformin for 3 days, and the amount of the viable cells was counted by the MTT-test. Data represent the mean value ± SD of three independent experiments. *p* < 0.05: $ versus MCF-7 cells, * versus control, # versus metformin-treated cells.

As is well known, the transferring of estrogen-dependent cells to steroid-free medium results in the complete suppression of ERα activity. To further analyze the role of ERα signaling in the realization of the cooperative effects of metformin, the subsequent experiments were performed on the MCF-7 cells cultured in the steroid-free medium (MCF-7/DCC cells). The analysis of the ERα activity revealed more than 20-fold suppression of the latter in the MCF-7/DCC cells (Figure 6a). The study of the efficiency of the combination metformin/rapamycin showed that MCF-7/DCC cells retained their sensitivity to growth-inhibitory metformin and/or rapamycin action (Figure 6b). Similarly, the parallel experiments on the estrogen-independent triple-negative MDA-MB-231 breast cancer cells showed that both metformin and rapamycin effectively inhibit cell growth, demonstrating the importance of ERα-independent ways in the realization of the cytostatic action of these drugs (Figure 6c).

### 2.4. Metformin Potentiates the ERα-Independent Tamoxifen Action

**Figure 7:**
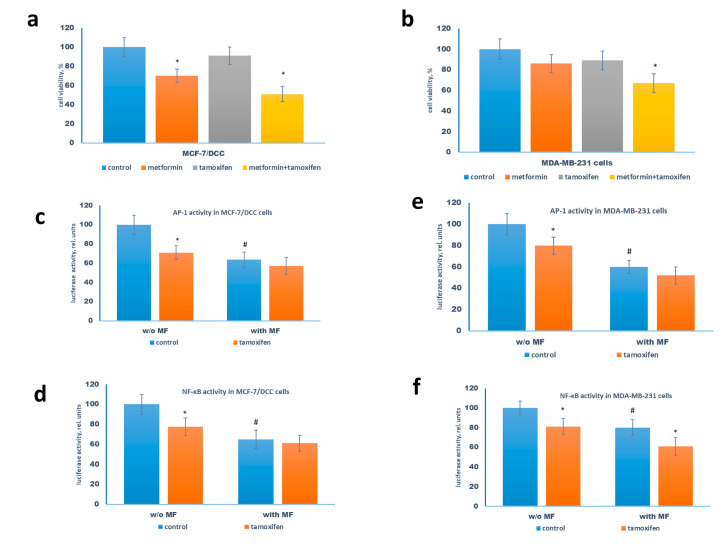
The sensitivity of MCF-7/DCC cells and MDA-MB-231 cells to tamoxifen. (**a**,**b**) Growth response. The cells were treated without or with 5 µM tamoxifen in the presence or absence 2 mM metformin for 3 days in phenol-free DMEM medium with 10% DCC (MCF-7/DCC cells) and in the standard DMEM medium with 10 % FCS, and the amount of the viable cells was counted by the MTT test. (**c**–**f**) Reporter assay. The cells were pretreated with or without 2 mM metformin for 2 days, then the cells were transfected with the AP-1 (**c,e**) or NF-κB (**d,f**) plasmid containing the luciferase reporter gene under the AP-1 or NF-κB-responsive elements, respectively, and β-galactosidase plasmid. Three hours after transfection the cells were treated with 2 mM metformin and 5 µM tamoxifen for 24 h. The luciferase and β-galactosidase activities were determined as described in Materials and Methods. Data represent the mean value ± SD of three independent experiments. *p* < 0.05: * versus respective control, # versus control cells w/o metformin.

The treatment of MCF-7/DCC and MDA-MB-231 cells with tamoxifen resulted in the slight inhibition of cell growth whereas the metformin co-treatment caused a remarkable increase in the cell sensitivity to tamoxifen (Figure 7a,b). The subsequent reporter analysis revealed the tamoxifen ability, alone or in combination with metformin, to inhibit the activity of AP-1 and NF-κB in both cell lines (Figure 7c–f); however, to a lesser extent than in MCF-7 cells (Figure 3).

**Figure 8:**
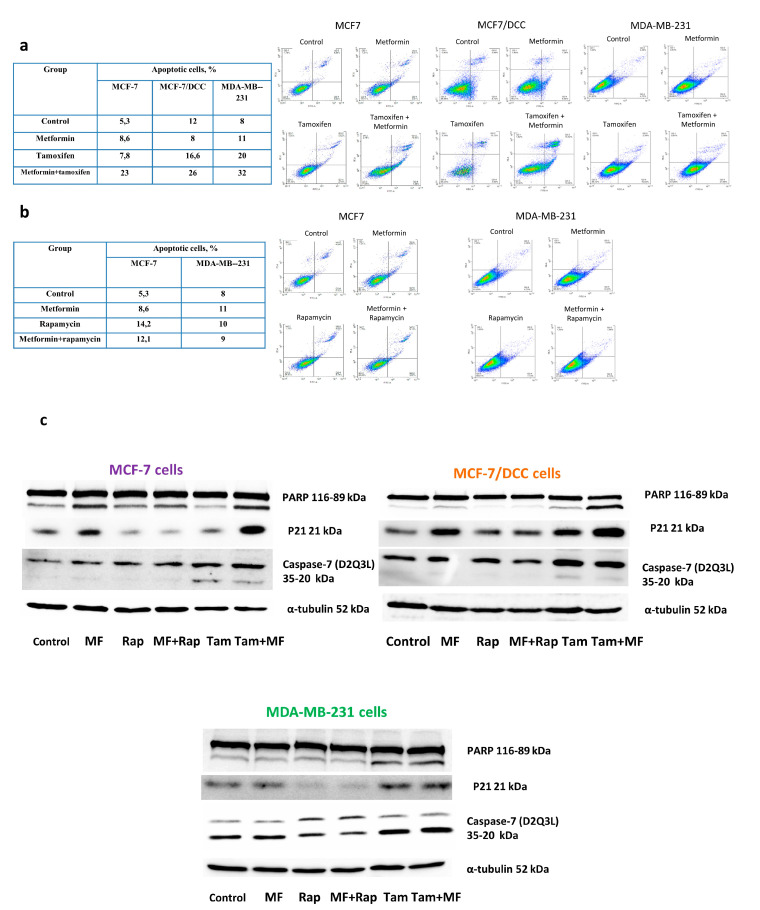
Tamoxifen and apoptotic cell death. The MCF-7, MCF-7/DCC, and MDA-MB-231 cells were treated with 1 µM rapamycin or 5 µM tamoxifen in the presence or absence of 2 mM metformin. Apoptosis was quantified via combined staining of annexin V and propidium iodide (**a**,**b**) as described in Methods (CV < 5%). Western blot analysis of PARP, p21/Waf, and caspase-7 was performed in the cell extracts. Caspase-7 (D2Q3L) antibody detects both the full-length (35 kDa—upper bands) and large (20 kDa—lower bands) subunits following caspase-7 activation. Protein loading was controlled by membrane hybridization with α-tubulin antibodies (**c**).

To further elucidate the mechanism of metformin sensitizing effects, the analysis of the apoptotic status of the treated cells was performed. The data of flow cytometric analysis showed the significant accumulation of the apoptotic cells under the metformin/tamoxifen co-treatment. The apoptotic effect of this drug combination was shown to be evident in both MCF-7 cells and the MCF-7/DCC and MDA-MB-231 cells (Figure 8a), and it was correlated with the PARP degradation and p21/Waf activation (Figure 8c). Caspase-7 cleavage was observed in the cells after tamoxifen or tamoxifen plus metformin treatments. Interestingly, active caspase-7 was also detected in the untreated MDA-MB-231 cells; this observation indicates the role of this caspase in the realization of cell death without inducers. Importantly, the separate treatment of the cells with tamoxifen or metformin resulted in the remarkable activation of apoptotic death (to a lesser extent for metformin) supporting the apoptotic potential of these drugs. On the contrary, rapamycin alone or in combination with metformin did not induce the apoptosis nether in the ERα+ MCF-7, nor in the ERα-negative MDA-MB-231 cells (Figure 8b). Because the cells with the inactive ERα, e.g., MCF-7/DCC and MDA-MB-231 cells, were still sensitive to apoptotic action of metformin/tamoxifen combination, the last data support the existence of the ERα-independent ways of apoptotic action of these drugs.

We conclude that the mechanism of the sensitizing effects of metformin depends on the nature of the tested drugs as well as on the type of tumor cells. In the case of rapamycin, the metformin increases its antiproliferative action, while under combination with tamoxifen, it induces apoptotic cell death. Moreover, tamoxifen apoptotic action is realized through the ERα-independent manner supporting the possible role of the combination of metformin/tamoxifen in the treatment of the therapy-resistant breast cancers.

## 3. Discussion

The key problem of the oncology is the high level of primary and/or acquired resistance of tumors to the antitumor drugs. Whereas multidrug resistance of cancer cells is based on the activation of ABC transporters responsible for the xenobiotic efflux, the resistance to the other drugs including target and hormonal agents may be mediated, at least in a part, via the rearrangement of the intracellular signaling network and compensatory activation of the signaling molecules [4,29]. Despite the extensive studies in this area, the investigation of the potential ways to overcome resistance is still a very significant and unresolved problem in molecular oncology.

One of the new approaches for overcoming resistance may be based on the usage of the compounds/inhibitors of the cell energetic metabolism. It was shown that energy deficiency provoked by the glucose deprivation and/or inhibition of glycolysis/gluconeogenesis may potentiate the antitumor drug activity. Similarly, the ability of bioenergetic inhibitors to increase cell sensitivity to the drugs was demonstrated in the tumor cells with a high level of primary or acquired resistance. However, the mechanism of such potentiating action of bioenergetic inhibitors and their action on cancer cells with different resistant phenotypes is still known not completely [20,23,30,31,32,33]. Only single observations demonstrate the possible targets of bioenergetic inhibitors—from the suppression of ABC transporters to activation of AMPK signaling [34,35,36].

Recently, it was found that metformin, the antidiabetic drug which affects the numerous metabolic parameters, exhibited antitumor activity via the activation of AMPK followed by the inhibition of mTOR signaling [15,37]. Moreover, the antigrowth effects of metformin may be based on the affecting of various cellular parameters, such as energy metabolism, rearrangement of pro- and antiapoptotic pathways, hypoxia-related pathways, oxidative stress, suppression of epithelial–mesenchymal transition, etc. [15,37]. Several studies demonstrate the additional inhibition of tumor growth under the combination of metformin with various cytostatic drugs [15,36,38]; however, the mechanism of such cooperative effects still not clear.

Here, we showed that relatively low concentrations of metformin (2 mM) result in a significant increase in the response of MCF-7 cells to cytostatic drugs: to a greater extent to antiestrogen tamoxifen, and to a lesser extent to mTOR inhibitor rapamycin. The analysis of the influence of drug combination on mTOR signaling revealed the marked inhibition of the phosphorylation of S6 kinase—one of the key downstream effectors of mTOR, that is correlated with inhibition of the activity of the transcription factors AP-1 and NF-κB. The combination of metformin and rapamycin did not cause apoptotic cell death, whereas cell co-treatment with metformin and tamoxifen resulted in the significant activation of apoptosis correlated with marked PARP degradation.

The sensitivity of the MCF-7 breast cancer cells to antiproliferative metformin action was demonstrated earlier by other authors [13,28,39] and in our publications [18,27]. High concentrations of metformin (5–10 mM) were shown to suppress the MCF-7 cell growth, both alone and in the combination with tamoxifen [27,40]. However, metformin’s influence on cell sensitivity to non-hormonal target drugs was not completely studied. We found that even low doses of metformin increase the MCF-7 cell response to both tamoxifen, via the stimulation the apoptotic cell death, and to rapamycin, via the suppression of mTOR -related signaling pathways. In general, our data are in agreement with the several observations demonstrating the additive anti-growth effect of metformin/rapamycin or metformin/tamoxifen in various tumor cells [22,23,27,40,41].

Another important question remains: Does the metformin co-treatment increase or restore the sensitivity of the resistant cells to cytostatic drugs? The results of our experiments have shown that the tamoxifen-resistant MCF-7/T and rapamycin-resistant MCF-7/Rap cells retained high sensitivity to the combination of metformin with the respective drugs and, similarly to the parent cells, respond to tamoxifen via the stimulation of cell death, and to rapamycin mainly via the mTOR suppression. Interestingly, despite the resistance of the MCF-7/T and MCF-7/Rap cells to growth inhibitory action of the respective drugs, the cells still respond to that via the suppression of the transcription factors AP-1 and NF-κB. Probably, it points at the activation of the compensatory regulatory mechanisms in the resistant cells controlling the expression of the growth-related genes, including the modification of the microRNA profile, changes in the methylation, acetylation, etc. [6,42,43].

Both tamoxifen and rapamycin inhibit estrogen receptor signaling that allowed us to proceed with the study of the latter in the realization of the metformin sensibilization effects.

We found that suppression of ERα activity under steroid depletion (MCF-7/DCC cells) does not attenuate the proapoptotic effect of metformin/tamoxifen co-treatment or the cytostatic effect of metformin/rapamycin treatment, demonstrating the ERα-independent mechanism of the metformin effects. Moreover, the results of the experiments with the ERα-negative MDA-MB-231 breast cancer cells revealed the marked suppression of cell growth and activation of apoptotic cell death under co-treatment with metformin and tamoxifen; moreover, even tamoxifen alone induced non-remarkable cell death supporting the existence of ERα-independent ways of tamoxifen action. Importantly, the tamoxifen ability to inhibit the growth of ERα-negative and/or resistant breast cancer cells was found in several observations [27,44,45,46,47,48]. We suggest that ERα-independent tamoxifen action may be realized via the suppression of growth-related or antiapoptotic transcription factors. The results of our study revealed the effect of tamoxifen-induced inhibition of AP-1 and NF-κB factors in the ERα-inactive cells (MCF-7/DCC cells, MDA-MB-231 cells) that may reflect the important role of these factors in the mediating of ERα-independent effects of tamoxifen.

In summary, we conclude that metformin co-treatment may be used for the increase and partial restoration of the cancer cell sensitivity to hormonal and target drugs. Moreover, the combination of metformin with tamoxifen induces the apoptotic death in the ERα-negative breast cancer cells opening the additional perspectives in the treatment of estrogen-independent breast tumors.

## 4. Materials and Methods

### 4.1. Cell Cultures and the Development of Drug-Resistant Sublines

The human breast cancer cell line MCF-7 (ATCC HTB-22™) was purchased from ATCC (Manassas, VA, USA). The cells were authenticated by morphology and STR profiling provided by Gordiz (Moscow, Russia) (http://gordiz.ru/). Metformin was purchased from the Tokyo Chemical Industry (Tokyo, Japan). Rapamycin and tamoxifen were produced by Cayman Chemical Company, Ann Arbor, MI, USA.

The rapamycin-resistant MCF-7/Rap and tamoxifen-resistant MCF-7/T sublines were established from the parent MCF-7 cells by long-term rapamycin or tamoxifen treatment, respectively. Briefly, MCF-7 cells were cultured in DMEM medium containing 1 µM rapamycin or 5 µM tamoxifen for 3 months, then the cells were transferred to rapamycin/tamoxifen-free medium, subsequent growth of sublines was maintained in the absence of the drugs. The MCF-7 cell line and the resistant MCF-7/Rap and MCF-7/T sublines were cultured in high glucose-containing (4.5 g/L) DMEM medium (PanEco) supplemented with 10% fetal bovine serum (FBS) (HyClone) at 37 °C and 5% CO_2_. To determine the cell response to compounds in drug combination studies the cells were treated with 1 µM rapamycin, 5 µM tamoxifen, and/or 2 mM metformin for 72 h in standard DMEM medium with 10% FBS, and the amount of the viable cells was assessed by the MTT test.

The cell growth was evaluated by the modified MTT (3-(4,5-dimethylthiazol-2-yl)-2,5-diphenyltetrazolium bromide) (Applichem) test [49] as described in [50]. Dose–response curves were analyzed by regression analysis using sigmoid curves (Log(concentration) vs. normalized absorbance). In this study, the half-maximal inhibitory concentration (IC_50_) values were determined using GraphPad Prism (Version 6.0).

Dimethylsulfoxide (DMSO) for the MTT test was obtained from Applichem. Ultrapure water for experiments was prepared by Milli-Q water purification system (Millipore).

### 4.2. Transient Transfection and Measurement of Reporter Gene Activity

For evaluation of the transcriptional activity of AP-1, NF-κB, and ERα, the cells were transfected with the plasmids containing luciferase reporter gene controlled by the respective responsive elements [51,52]. The transfection was carried out for 4 h at 37 °C using Metafectene PRO (Biontex, München, Germany). To this end, Metafectene PRO (0.8 µL) was complexed with 0.4 µg of DNA to transfect one well (24 well plate, Corning, Corning, NY, USA). To control the efficiency and potential toxicity of the transfection, the cells were transfected with the β-galactosidase plasmid. All subsequent experiments were performed in 24 h after transfection. The luciferase activity was measured according to a standard protocol (Promega, Madison, WI, USA) using a Tecan Infinite M200 Pro, and calculated in arbitrary units as the ratio of the luciferase/galactosidase activity as described in [53].

### 4.3. Western Blot Analysis of Cell Lysates

For immunoblotting, the cells at the 80% monolayer stage were lysed in 150 μL buffer: 50 mM Tris-HCl pH 7.4; 1% Igepal CA-630; 150 mM NaCl; 1 mM ethylenediamine tetraacetate; 1 mM dithiothreitol; 1 μg/mL aprotinin, leupeptin, and pepstatin; and 1 mM sodium fluoride and sodium orthovanadate. Samples were incubated on ice for 20 min before centrifugation (10,000 *g*, 10 min, 4 °C). Electrophoresis was performed with a 10% polyacrylamide gel followed by protein transfer to a nitrocellulose membrane and immunoblotting. Protein content was determined by the Bradford method.

Cell lysates (40 µg protein) were separated by 10% SDS-PAGE under reducing conditions, transferred to a nitrocellulose membrane (SantaCruz, Santa Cruz, CA, USA), and processed according to the standard protocol. To prevent nonspecific absorption, the membranes were treated with 5% nonfat milk (AppliChem) solution in TBS buffer (20 mM Tris, 500 mM NaCl, pH 7.5) with 0.1% Tween-20 and then incubated with primary antibodies overnight at +4 °C.

Primary antibodies to (phosphorylated) Akt, (phosphorylated) mTOR, (phosphorylated) S6 kinase, (phosphorylated) AMPK, PARP, p21/Waf, and caspase-7 (Cell Signaling Technology, Danvers, MA, USA) were used; the antibodies against α-tubulin (Cell Signaling Technology, Danvers, MA, USA) were used to standardize loading. Appropriate IgGs (Jackson ImmunoResearch, Cambridgeshire, UK) conjugated with horseradish peroxidase were used as secondary antibodies. Signals were detected by ECL reagent prepared as described in Mruk’s protocol [54] and the ImageQuant LAS4000 system for chemiluminescence (GE HealthCare, Chicago, IL, USA).

### 4.4. Apoptosis Measurement

Cells were treated with tested drugs for 3 days. Apoptosis was quantified via combined staining of annexin V and propidium iodide (PI) using the Annexin V–FITC Kit (Molecular probes, Waltham, MA, USA) according to the manufacturer’s protocol. The FITC and PI fluorescence was measured on a NovoCyte 2000R flow cytometer (ACEA Biosciences, San Diego, CA, USA), and the percentages of apoptotic cells were analyzed using NovoExpress v.1.2.4 software.

### 4.5. Statistical Analysis

Each experiment was repeated three times with three technical replicates. Statistical analysis was performed using Microsoft Excel and Statistica. Results were expressed as mean ± SD (standard deviation value) if not stated explicitly. *p*-value of <0.05 was used as statistically significant.
